# LncRNA-NEAT1 blocks the Wnt/β-catenin signaling pathway by targeting miR-217 to inhibit trophoblast cell migration and invasion

**DOI:** 10.1007/s10815-024-03124-7

**Published:** 2024-05-06

**Authors:** Ling-ling Jiang, Dan-lin Yang, Qing Han, Hua-le Zhang, Mian Pan, Jian-ying Yan

**Affiliations:** Fujian Maternity and Child Health Hospital, College of Clinical Medicine for Obstetrics & Gynecology and Pediatrics, Fujian Medical University, Fuzhou, 350001 China

**Keywords:** Preeclampsia, Trophoblast cells, MicroRNA-217, lncRNA NEAT1, Cell invasion, Cell migration

## Abstract

**Objective:**

This study aimed to study the correlation between preeclampsia (PE) and lncRNA nuclear paraspeckle assembly transcript 1 (NEAT1), and to examine the molecular mechanisms behind the development of PE.

**Methods:**

30 PE and 30 normal pregnant women placental samples were assessed the levels of NEAT1 and miR-217 by quantitative real-time PCR (qRT-PCR). The trophoblast cell line HTR8/SVneo was used for silencing NEAT1 or miR-217 inhibitor in the absence or presence of an inhibitor and H_2_O_2_. Cell counting Kit 8 (CCK-8), flow cytometry, and Transwell were used to detect cell proliferation, apoptosis, migration, and invasion. Luciferase reporter gene assay was utilized to verify the binding between miR-217 and Wnt family member 3 (Wnt3), and between the miR-217 and NEAT1. Proteins related to the Wnt/β-catenin signaling pathway were detected using western blotting.

**Results:**

The PE group exhibited a significantly downregulated expression of miR-217 and a significantly upregulated expression of NEAT1. NEAT1 targeted miR-217, and Wnt is a miR-217 target gene. siRNA-NEAT1 inhibited the apoptosis of trophoblast cells, but promoted their invasion, migration, and proliferation. MiR-217 inhibitor could partially reverse the effects of siRNA-NEAT1. The expression of the Wnt/β-catenin signaling pathway-related proteins, WNT signaling pathway inhibitor 1 (DKK1), cyclin-D1 and β-catenin, was significantly increased after siRNA-NEAT1.

**Conclusions:**

NEAT1 could reduce trophoblast cell invasion and migration by suppressing miR-217/Wnt signaling pathway, leading to PE.

## Introduction

Preeclampsia (PE), a hypertensive disorder occurring only during pregnancy, is one of the prime reasons for infant and maternal morbidity and mortality [1]. PE is a disease caused by multiple factors, multiple pathways, multiple mechanisms. However, the mechanisms underlying PE are still not completely understood. Insufficient remodeling of the trophoblast endometrial spiral artery is one of the main recognized pathogenic factors associated with PE [2]. Trophoblasts can remodel the uterine spiral artery by their ability to differentiate, migrate, proliferate, invade, and induce apoptosis [2].

Long noncoding RNAs (lncRNAs) cannot encode proteins and are shorter than 200 bp in length. They not only participate in cell proliferation, apoptosis, angiogenesis, and tumor metastasis but also regulate microRNAs (miRNAs) with the same noncoding properties to indirectly affect the target genes of miRNAs and play relevant physiological roles [3]. Studies have shown that some lncRNAs can regulate trophoblasts and participate in the onset of PE [4, 5]. LncRNA nuclear-enriched abundant transcript-1 (NEAT1) expression in the placenta of fetuses with intrauterine growth restriction was higher than that of fetuses without intrauterine growth restriction at term [6], suggesting that NEAT1 may have a major role in pregnancy. However, the role of NEAT1 in the onset of PE has not been fully elucidated.

MiRNAs are small noncoding RNAs that regulate target gene expression at the post-transcriptional level. MiRNAs are expressed widely in a large variety of cells [7]. Due to the abundance of miRNA binding sites on RNA transcripts, miRNAs can regulate gene expression and interact with one another by engaging in competitive binding, acting as competitive endogenous RNAs (ceRNAs) [8]. MiR-217 is localized on chromosome 2p16.1, and plays an essential role in tumorigenesis [9, 10]. By regulating the biological axis of miR-217/mitogen-activated protein kinase 1 (MAPK1), lncRNA colorectal neoplasia differentially expressed (cRNDE) could induce hepatocellular carcinoma cells invasion, migration and proliferation [9]. MiR-217 could inhibit gastric cancer progression and metastasis by regulating enhancer of zeste 2 polycomb repressive complex 2 subunit (EZH2) [10]. Wnt family member 3 (Wnt3) is a key signaling protein in the Wnt signaling pathway and is crucial in tumor development and progression [11]. Silencing of lncRNA SPRY14-IT1 in extravillous trophoblasts downregulated Wnt3 and Wnt family member 5B (Wnt5b) expression in vitro [4].

In this study, we found that NEAT1 targeted miR-217 by using bioinformatics software, and Wnt3 was a potential target gene for miR-217. Subsequently, their role in PE was further validated through in vitro cell experiments. Our findings provide new insights and directions for the research and treatment of PE.

## Materials and methods

### Collection of placental samples

Thirty PE (PE group) and 30 normal (Normal group) pregnant Chinese women were included in this study. None of them had a hypertensive emergency. All the women were delivered between January 2019 to January 2020 in the Fujian Maternity and Child Health Hospital. As previously described [12], PE was diagnosed when a pregnant woman had hypertension for the first time after 20 weeks of pregnancy, and the hypertension was accompanied by at least one of the following symptoms: urinary protein ≥ 300 mg/24 h, or random urinary protein 1 + , urinary protein/creatinine ≥ 0.3.

All the participants were delivered by cesarean section. Normal pregnant women were delivered by cesarean section for one of the following reasons: abnormal fetal position, abnormal birth canal, previous history of cesarean section, or social factors. None of the pregnant women underwent trial labor, and there were no symptoms of clinical infection or premature rupture of membranes. This study excluded pregnant women who suffered from other pregnancy complications but included patients with singleton pregnancies.

Placental tissue was immediately collected from the root of the umbilical cord after the delivery, and areas of hemorrhage, infarction and calcification were avoided during tissue collection. Placenta was subdivided under aseptic condition into 1.0 cm × 1.0 cm × 1.0 cm pieces. The placental samples were rinsed with cold saline, snap frozen in liquid nitrogen, and then stored in a refrigerator at -70 °C until further use.

### Ethical statement

The study was performed in line with the principles of the Declaration of Helsinki, and the rights of all participants were protected. This study obtained informed consent from each participant. Approval was granted by the Ethics Committee of Fujian Maternity and Child Health Hospital (2019–115).

### Cell culture

The trophoblast cell line, HTR8/SVneo cells, purchased from China Beina Biological Company, were cultured at 37℃ with 5% CO_2_ and 20% O_2_ in the RPMI-1640 complete medium (Gibco, Thermo Fisher Scientific, Waltham, USA) containing calf serum (10%, Gibco, Thermo Fisher Scientific, Waltham, USA) and 100 U/mL of streptomycin and penicillin for two or three days until 80% confluence. Cell passage was performed by enzyme digestion. HTR8/SVneo cells were subcultured and inoculated at a cell density of 1 × 10^5^cells/mL. Additionally, HTR8/SVneo cells were treated with 175 µM H_2_O_2_ (Sigma-Aldrich, St. Louis, MO) for 48 h at 37 °C in a 5% CO_2_ and 20%O_2_ incubator.

### Quantitative real-time PCR (qRT-PCR)

Total RNA was extracted from HTR8/SVneo cells and placental tissues by TRIzol reagent (Invitrogen, Carlsbad, CA, USA). Then Prime ScriptTM RT reagent Kit with gDNA Eraser (Takara, Shiga, Japan) was used to reverse RNA. cDNA was used as a template to assay the expression level for both miR-217 and lncRNA-NEAT1 in the HTR8/SVneo cells and placental tissues using the following primers: NEAT1 (forward, 5’- GCAGATCAGCATCCTTCG -3’; reverse, 5’- ACAAGACACCTGTGACAAATG-3’), glyceraldehyde-3-phosphate dehydrogenase (GAPDH) as an internal control for NEAT1 (forward, 5’-GGGAAACTGTGGCGTGAT -3’; reverse, 5’- GAGTGGGTGTCGCTGTTGA-3’), miR-217 (forward, 5’-GGGGTACTGCATCAGGAACTG-3’; reverse, 5’-AACTGGTGTCGTGGAGTCGGC-3’), and U6 as an internal control for miR-217 (forward, 5’- CTCGCTTCGGCAGCACA-3’; reverse, 5’-AACGCTTCACGAATTTGCGT-3’). QRT-PCR was conducted using a PCR system (Roche LightCycler 480). Data were analyzed using the comparative Ct method (2^−ΔΔCt^) with GAPDH as an internal control.

### Cell transfection

HTR8/SVneo cells passaged to the third generation were inoculated into a 12 well plate until 70%–80% confluence. Then the cells were transfected with miR-217 mimics, mimic control, siRNA, siRNA-NEAT1, miR-217 inhibitor or inhibitor control using Lipofectamine 2000 (Invitrogen, USA) according to the manufacturer's instructions. After 24 h, the transfection medium was replaced with fresh HTR8/SVneo cell culture medium. After 48 h of cell culture, subsequent experiments were conducted.

### Cell proliferation analysis

HTR8/SVneo Cells (3000 cells/well) seeded in 96-well plates containing 100 µL of culture medium per well were incubated (5% CO_2_, 95% O_2_, 37 ℃) overnight. They were then divided into different groups and re-cultured for 48 h. Afterwards, 10 µ L of Cell Counting Kit-8 (CCK-8) reagent (Tongren, Japan) was added to each well, followed by four-hour incubation. The absorbance value of each well was then calculated using a microplate reader (Thermo Fisher Scientific, Waltham, USA) at 450 nm.

### Transwell assay

Before inoculation, cells were rinsed with phosphate buffered saline (PBS) for five minutes, digested, and then washed in serum-free medium (opti-MEM, Gibco,Thermo Fisher Scientific, Waltham, USA). The cells were then resuspended in 1% fetal bovine serum (FBS) medium and seeded on a Transwell plate after being diluted to 1 × 10^5^ cells/mL. Each chamber received 0.5 mL of cell suspension, whereas the lower chamber received 0.75 mL of growth medium containing 10% FBS. The cells were then cultivated for two days (48 h) in a 37 °C incubator. After removing the cells from the growth medium, they were rinsed with 1 mL of PBS, fixed with 1 mL of 4% formaldehyde solution, and maintained at room temperature for 20 min. The fixation solution was entirely aspirated and the cells were washed once with PBS again. Subsequently, the cells were stained with 1 mL of 0.5% crystal violet solution and dried for 30 min. After three washes with PBS, non-migrated cells inside the Transwell chamber were removed and counted under a 200 × light microscope.

### Invasion assay

Serum-free medium (opti-MEM, Gibco,Thermo Fisher Scientific, Waltham, USA) was used to replace cell medium in each group 24 h before the experiment. Eighty microliters of Matrigel (50 mg/L, 1: 8 dilution) was added to the Transwell upper chamber, and the Transwell plate was put in a 37 °C incubator for 30 min. Before resuspending the cells and plating them in the Transwell plate, they were counted and diluted to 1 × 10^5^ cells/mL in the medium containing 1% FBS. After that, each chamber received 0.5 mL of cell suspension, whereas the lower chamber received 0.75 mL of culture medium containing 10% FBS. On completion of incubation for two days (5% CO_2_, 20% O_2_, and 37 °C), the cells were removed from the growth medium, rinsed with 1 mL PBS, fixed in 1 mL of 4% formaldehyde solution, and maintained at room temperature for 20 min. After aspiration of the fixation solution, the cells were stained with 1 mL of 0.5% crystal violet solution and dried for 30 min. The non-invasive cells were thoroughly wiped away with a cotton swab before viewing the plate under a 200 × microscope.

### Apoptosis assay

Flow cytometry was utilized to determine whether lncRNA-NEAT1 could affect the apoptosis of HTR8/SVneo cells. A total of 1 × 10^6^ cells were resuspended in a medium, centrifuged twice at 400 g (4 °C, 5 min). The supernatant was then discarded and the cells were resuspended in 200 µL of PBS before being treated with 10 µL Annexin V-FITC (fluorescein-isothiocyanate) and propidium iodide (PI) (Becton, Dickinson and Company, USA). The cells were then gently mixed and incubated for 30 min at 4 °C away from light. Cell apoptotic rates were evaluated by FACSan flow cytometry (BD Biosciences, San Jose, CA, USA).

### Dual-luciferase reporter gene assay

The constructed NEAT1-mutant (NEAT1-Mut), NEAT1-wild type (NEAT1-WT), mimic-NC, or miR-217-5p mimic luciferase reporter plasmid (Beyotime Biotechnology, China) was transfected into HTR8/SVneo cells. Moreover, the constructed Wnt3-Mut or Wnt3-WT luciferase reporter (Yesen Biotechnology, China) plasmid was co-transfected into HTR8/SVneo cells with mimic-NC or miR-217-5p mimic. After 48 h of transfection, the luciferase activity was measured by dual luciferase reporter gene assay system.

### Western blotting

The cells of each group were collected and completely lysed by adding protein lysis solution. The cells were then centrifuged at 12 000 g/min for 20 min at 4 ℃, and the supernatant was transferred to a new eppendorf tube. The protein concentration was determined by bicinchoninic acid (BCA) kit. After denaturation by boiling, each group was subjected to sodium dodecyl sulfate polyacrylamide gel electrophoresis (SDS-PAGE) electrophoresis by adding the same mass of protein supernatant, followed by protein wet transfer onto the polyvinylidene fluoride (PVDF) membranes. The membranes were then blocked with 5% bovine serum albumin (BSA) at room temperature for two hours, and incubated overnight at 4 ℃ with WNT signaling pathway inhibitor 1 (DKK1, ab307367, Abcam, UK), Cyclin-D1 (ab16663, Abcam, UK), β-catenin (ab68183, Abcam, UK) and β-actin (ab115777, Abcam, UK) primary antibody dilutions. Afterwards, the membranes were incubated with horseradish peroxidase-conjugated anti-rabbit (ab205718, Abcam, UK) or anti-mouse (ab205719, Abcam, UK) immunoglobulin G (IgG) for one hour at room temperature. The protein bands were exposed by enhanced chemiluminescence and analyzed using ImageJ software.

### Statistical analysis

All experiments were repeated three times. Statistical Package for the Social Science (SPSS) was used for all statistical analyses. Data were presented as mean ± standard error of mean (SEM), with comparison tests including t-test (for two groups), one way analysis of variance (ANOVA) (for three or more groups), and Newman-Keuls post hoc test (or Bonferroni post hoc test). Statistically significance was only meaningful at *P* < 0.05.

## Results

### Clinical indicators in PE and normal pregnant women

General characteristics were collected from pregnant women (Table 1), PE and normal pregnant wo men showed no significant difference in the age of participants, gestational age, and pre-pregnancy or pregnancy body mass index. However, the systolic and diastolic blood pressure in the PE group was significantly higher than that of the normal group.

**Table 1:** Relevant clinical indicators in pregnant women (* *p* < 0.05)

group	case	age (years)	gestational age (weeks)	pre-pregnancy BMI (Kg/m2)	pregnancy BMI (Kg/m2)	blood	pressure
						Systolic pressure	Diastolic pressure
PE group	30	30.7 ± 5.5	37.7 ± 1.4	21.3 ± 2.4	26.7 ± 1.9	155.9 ± 10.8*	99.2 ± 6.8*
Normal group	30	30.0 ± 4.7	38.3 ± 1.3	20.4 ± 1.7	25.8 ± 1.9	116.2 ± 6.3	73.8 ± 6.6

### Expression of NEAT1 in placental tissues

**Fig. 1:**
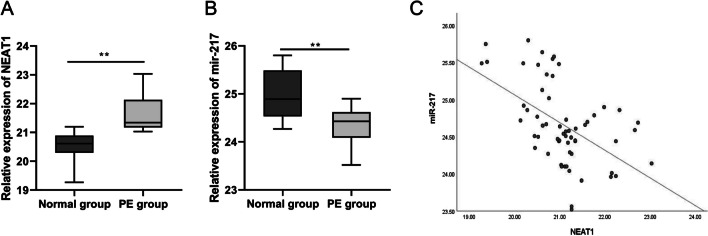
Expression of NEAT1 in placental tissues. The expression of NEAT1 (**A**) and miR-217 (**B**) in the placenta of the PE and normal pregnant women as detected by qRT-PCR. (**C**) The correlation analysis between NEAT1 and miR-217 in the placenta of the PE group. T-Test was used for data analysis. Data were presented as mean ± SEM. **p ≤ 0.01 vs. Normal group

The qRT-PCR findings revealed that PE women had significantly higher NEAT1 and significantly lower miR-217 expression than normal pregnant women (Fig. 1A and B). NEAT1 and miR-217 expression in PE women were negatively correlated (Fig. 1C).

### Effects of NEAT1 on the proliferation, migration and invasion of trophoblast cells

**Fig. 2:**
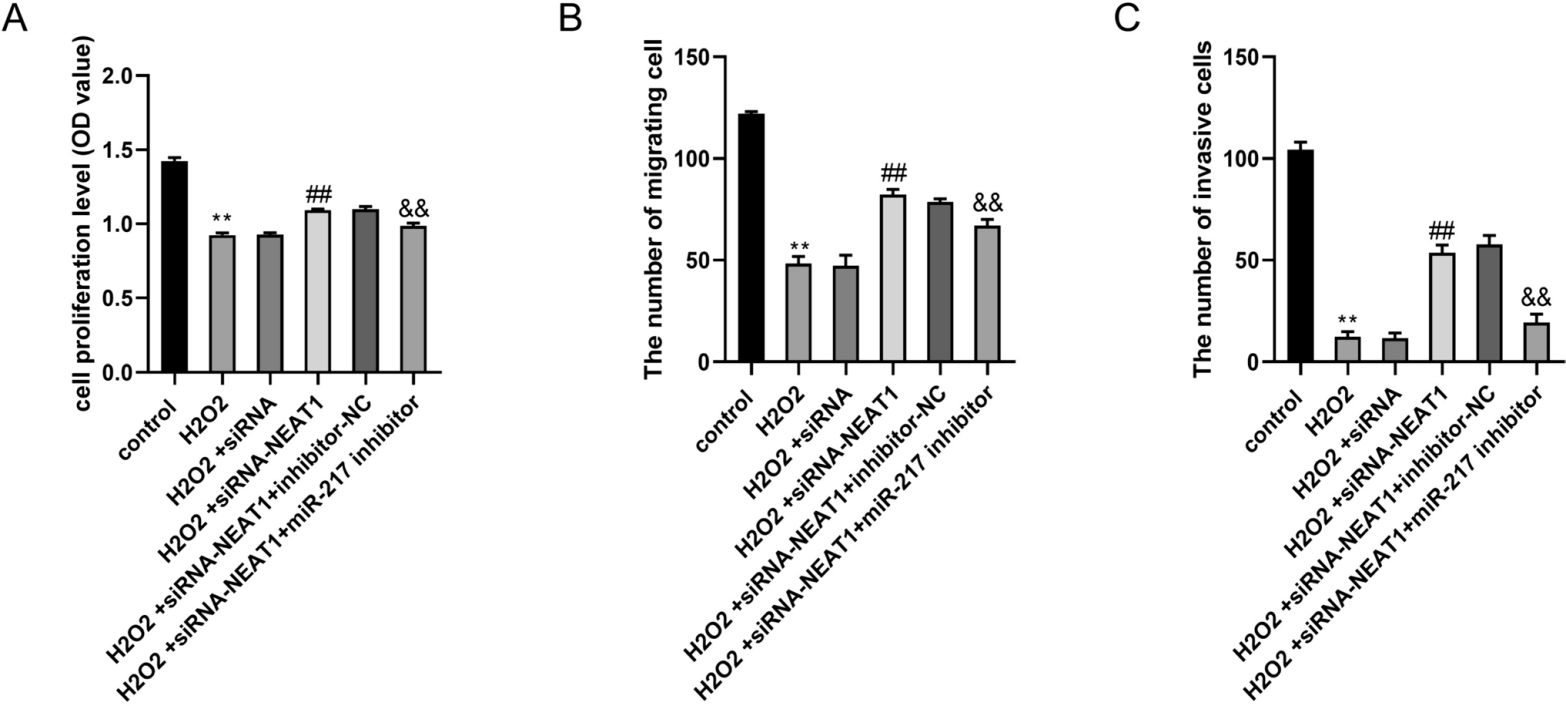
Effects of NEAT1 on the proliferation, migration and invasion of trophoblast cells (**A**) The average measured value of each group at 450 nm. (**B**) The results of the cell migration experiment. (**C**) The results of the cell invasion experiment. One way ANOVA and Bonferroni post hoc test were used for data analysis. Data were presented as mean ± SEM. **p ≤ 0.01 vs. Control group, ##*p* ≤ 0.01 vs. H_2_O_2_ + siRNA group, &&*p* ≤ 0.01 vs. H_2_O_2_ + siRNA-NEAT1 + inhibitor-NC group

To discover the effects of NEAT1 and miR-217 on the invasion, proliferation and migration of trophoblast cells, HTR8/SVneo cells were divided into six groups: the control group, H_2_O_2_ group, H_2_O_2_ + siRNA group, H_2_O_2_ + siRNA-NEAT1 + inhibitor-NC group, H_2_O_2_ + siRNA-NEAT1 group and H_2_O_2_ + siRNA-NEAT1 + miR-217 inhibitor group. The CCK-8 assay revealed that H_2_O_2_ significantly inhibited cell proliferation (*p* < 0.05 vs. control group), and siRNA-NEAT1 promoted cell proliferation (*p* < 0.05 vs. H_2_O_2_ + siRNA group). The cell proliferation level in the H_2_O_2_ + siRNA NEAT1 + miR-217 inhibitor group was significantly lower than that in the H_2_O_2_ + siRNA NEAT1 + inhibitor NC group (Fig. 2A). The migration and invasion of HTR8/SVneo cells, as detected by Transwell assays, were inhibited by H_2_O_2_ but promoted by siRNA-NEAT1. In addition, the cell migration and invasion level in the H_2_O_2_ + siRNA NEAT1 + miR-217 inhibitor group was significantly lower than that in the H_2_O_2_ + siRNA NEAT1 + inhibitor NC group (Fig. 2B, C).

### Effects of NEAT1 on trophoblast cell apoptosis

**Fig. 3:**
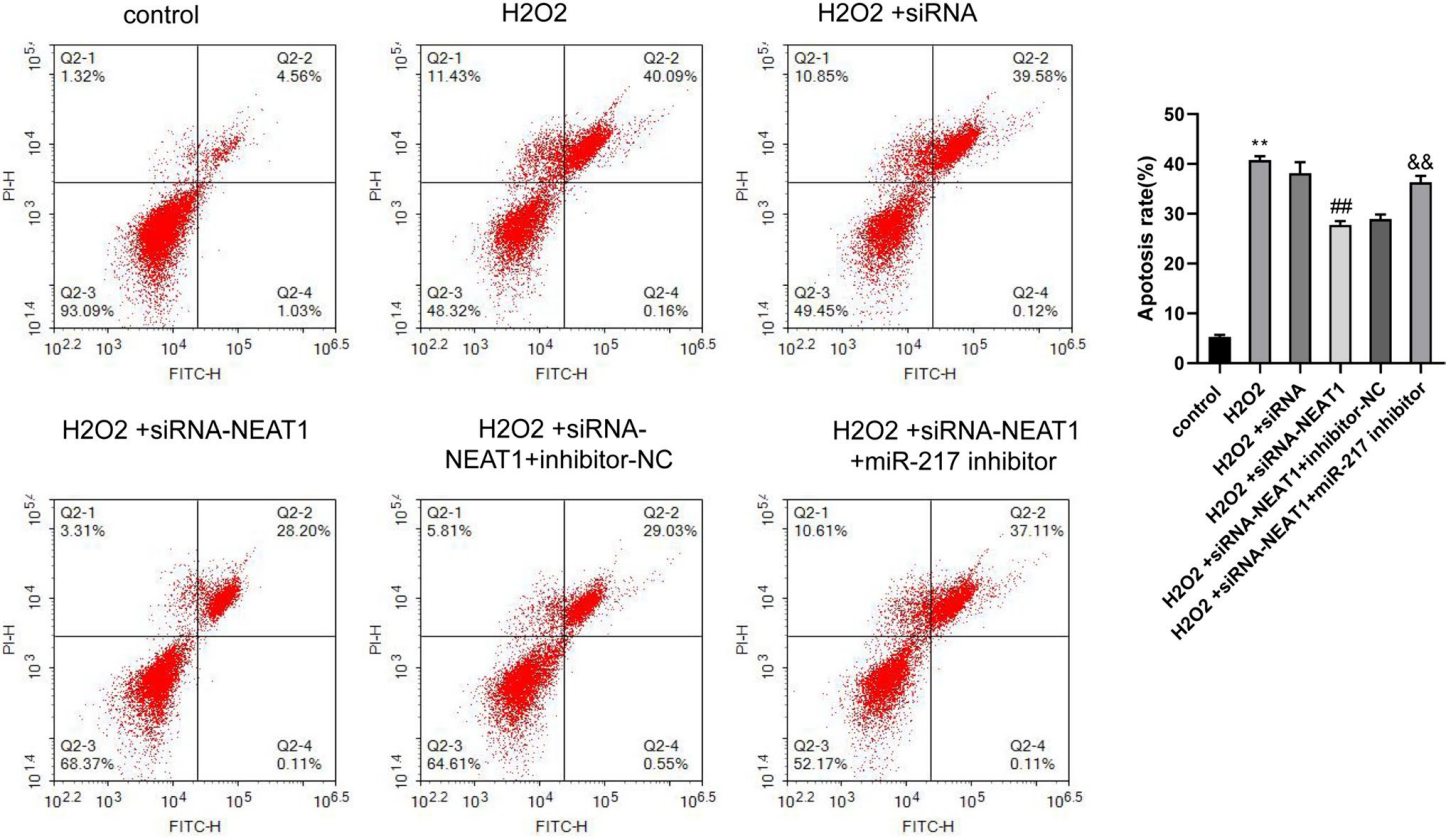
Effects of NEAT1 on trophoblast cell apoptosis. The apoptosis was detected by flow cytometry. One way ANOVA and Bonferroni post hoc test were used for data analysis. Data were presented as mean ± SEM. **p ≤ 0.01 vs. Control group, ##*p* ≤ 0.01 vs. H_2_O_2_ + siRNA group, &&*p* ≤ 0.01 vs. H_2_O_2_ + siRNA-NEAT1 + inhibitor-NC group

HTR8/SVneo cells were divided into the six groups described above, and their apoptosis in each group was evaluated by flow cytometry. H_2_O_2_ significantly promoted the cell apoptosis ability (*p* < 0.05 vs. control group), siRNA-NEAT1 inhibited cell apoptosis (*p* < 0.05 vs. H_2_O_2_ + siRNA group), and miR-217 inhibitor could partially reverse the effect of siRNA-NEAT1 (*p* < 0.05 vs. H_2_O_2_ + siRNA-NEAT1 + inhibitor-NC group) (Fig. 3).

### The results of dual luciferase reporter gene analysis

**Fig. 4:**
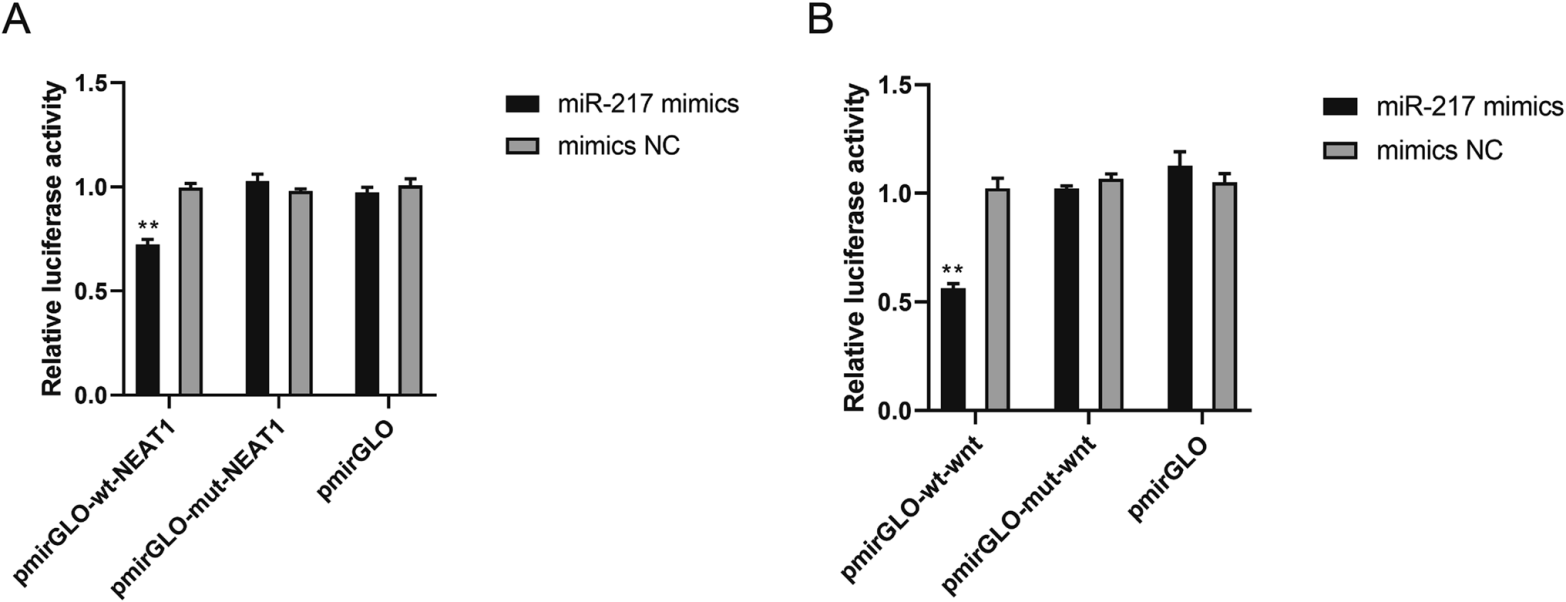
The results of dual luciferase reporter gene analysis. (**A**) The expression of relative luciferase activity, and effect of miR-217 on luciferase reporter gene activity of NEAT1 mRNA 3'UTR. (**B**) The expression of relative luciferase activity, and effect of miR-217 on luciferase reporter gene activity of Wnt3 mRNA 3'UTR. T-Test was used for data analysis. Data were presented as mean ± SEM. *p ≤ 0.05 vs. Mimics NC group

By comparing to the mimics NC group, the luciferase activity of miR-217 mimics’ had a significant decline in the pmirGLO-wt-NEAT1 group. However, its luciferase activity had no significant changes after transfection with the recombinant vector plasmid pmirGLO-mut-NEAT1. The result after transfection with the blank vector plasmid pmirGLO was as the same as the pmirGLO-mut-NEAT1 group, with no notable changes (Fig. 4A). The dual luciferase reporter gene analysis suggested that compared with the mimics NC group, the luciferase activity of Wnt3 mRNA 3'UTR, specifically Wnt3-WT, in the miR-217 mimic group was obviously declined. However, Wnt3-Mut mRNA 3'UTR had no significant difference on its luciferase activity between the mimics NC and miR-217 mimics groups (Fig. 4B).

### The expression of Wnt/β-catenin signaling pathway-related factors in placental trophoblast cells

**Fig. 5:**
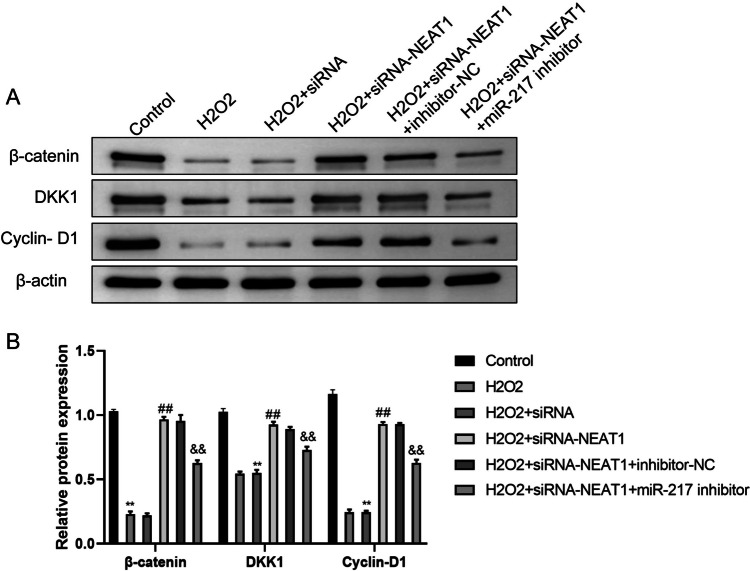
The expression of wnt/β-catenin signaling pathway-related factors in placental trophoblast cells. Expression of proteins related to the Wnt/β-catenin signaling pathway in trophoblast cells of each group was compared. One way ANOVA and Bonferroni post hoc test were used for data analysis. Data were presented as mean ± SEM. **p ≤ 0.01 vs. Control group, ##*p* ≤ 0.01 vs. H_2_O_2_ + siRNA group, &&*p* ≤ 0.01 vs. H_2_O_2_ + siRNA-NEAT1 + inhibitor-NC group

Western blotting was responsible for assessing the Wnt/β-catenin signaling pathway-related factors, like the proteins (Cyclin-D1, DKK1 and β-catenin) in HTR8/SVneo cells. Compared with the control group, the protein levels of β-catenin, DKK1, and Cyclin D1 were significantly reduced in the H_2_O_2_ group. Compared with the H_2_O_2_ + siRNA group, the protein levels of these three factors were significantly increased in the H_2_O_2_ + siRNA NEAT1 group. Compared with the H_2_O_2_ + siRNA NEAT1 + inhibitor NC group, the protein levels of these three factors were significantly reduced in the H_2_O_2_ + siRNA NEAT1 + miR-217 inhibitor group. There was no statistically significant difference between the H_2_O_2_ group and H_2_O_2_ + siRNA group, and between the H_2_O_2_ + siRNA NEAT1 group and H_2_O_2_ + siRNA NEAT1 + inhibitor NC group (Fig. 5).

## Discussion

As a common and dangerous pregnancy complication, PE is associated with high maternal and infant morbidity and mortality during the perinatal period [13]. Inadequate remodeling of the spiral uterine artery, a mechanism contributing to the onset of PE, can result from inadequate placental trophoblast migration and invasion capacity [13]. Researches have shown that some lncRNAs can control the function of trophoblast cells, and are involved in the progression of PE [4, 5]. We investigated the mechanism of that NEAT1 affected the migration, apoptosis, invasion and proliferation of trophoblast cells by the miR-217/Wnt3 axis. The findings of this research may open a new perspective for the therapy of PE.

LncRNA NEAT1 is a novel lncRNA discovered in recent years, and its normal expression is related closely to apoptosis and cell cycle of tumor cells [14]. Placental development and tumor progression share many features. For the patients suffering from PE, NEAT1 expression has been found to express a high level in the placenta [14]. Many cellular processes involved in placental development, including cell metabolism, migration, and trophoblast differentiation, are controlled by miRNAs [15, 16].

In the study at tissue level, we made a comparison between the characteristics of the normal pregnant women and pregnant women with PE, and discovered that the expression level of miR-217 in patients with PE was dropped, whereas the level of NEAT1 was increased. Thus, NEAT1 might target miR-217 to cause placental dysplasia, which was further confirmed by dual luciferase reporter gene assay.

In addition, this study found that H_2_O_2_ inhibited the migration, proliferation, and invasion of HTR8/SVneo cells, but promoted HTR8/SVneo cell apoptosis. Interference with NEAT1 could enhance migration, proliferation, and invasion of HTR8/SVneo cells but suppressed their apoptosis. However, the effects of siRNA-NEAT1 could be partly reversed by miR-217 inhibitor. These results suggested that NEAT1 inhibits the invasion and migration of placental trophoblast cells by targeting miR-217.

The Wnt/β-catenin signaling pathway is an essential pathway associated with cancer progression [17]. It has been reported that membrane-associated β-catenin and Wnt3 can promote the development of progenitor trophoblast in human blastocysts [18]. In this study, the potential target of miR-217 was predicted by the bioinformatics software, and the binding site of miR-217 was found to be located on the 3'UTR of Wnt3 gene. Wnt3 was confirmed as a target gene of miR-217 by the dual luciferase reporter gene assay. SiRNA-NEAT1 increased β-catenin, DKK1 and cyclin-D1. Collectively, NEAT1 can significantly block Wnt/β-catenin signaling pathway, but this mechanism of action will be inhibited by miR-217.

## Conclusion

In conclusion, the expression of miR-217 in the placenta of patients with PE was reduced, whereas the expression of NEAT1 was elevated. NEAT1 blocks the Wnt/β-catenin signaling pathway by targeting miR-217 to inhibit trophoblast cell migration and invasion. These findings may provide a novel perspective for the treatment of PE.

## Data Availability

The dataset supporting this article is available from the corresponding author.
